# Developing 9,10-anthracene Derivatives: Optical, Electrochemical, Thermal, and Electrical Characterization

**DOI:** 10.3390/ma12172726

**Published:** 2019-08-26

**Authors:** Mikhail Y. Vorona, Nathan J. Yutronkie, Owen A. Melville, Andrew J. Daszczynski, Kwame T. Agyei, Jeffrey S. Ovens, Jaclyn L. Brusso, Benoît H. Lessard

**Affiliations:** 1 Department of Chemical and Biological Engineering, University of Ottawa, 161 Louis Pasteur, Ottawa K1N 6N5, ON, Canada; 2 Department of Chemistry and Biomolecular Sciences, University of Ottawa, 150 Louis Pasteur, Ottawa K1N 6N5, ON, Canada; 3 X-Ray Core Facility, University of Ottawa, 150 Louis Pasteur, Ottawa K1N 6N5, ON, Canada

**Keywords:** OTFTs, anthracene, crystal, thin film, transistor, packing, semiconductor, Suzuki-Miyaura cross-coupling reaction

## Abstract

Anthracene-based semiconductors are a class of molecules that have attracted interest due to their air stability, planarity, potential for strong intermolecular interactions, and favorable frontier molecular orbital energy levels. In this study seven novel 9,10-anthracene-based molecules were synthesized and their optical, electrochemical, and thermal properties were characterized, along with their single crystal arrangement. We found that functionalization of the 9,10-positions with different phenyl derivatives resulted in negligible variation in the optical properties with minor (±0.10 eV) changes in electrochemical behavior, while the choice of phenyl derivative greatly affected the thermal stability (*T*_d_ > 258 °C). Preliminary organic thin film transistors (OTFTs) were fabricated and characterized using the 9,10-anthracene-based molecules as the semiconductor layer. These findings suggest that functionalization of the 9,10-position of anthracene leads to an effective handle for tuning of the thermal stability, while having little to no effect on the optical properties and the solid-state arrangement

## 1. Introduction

Organic electronic materials, namely organic light emitting diodes (OLEDs), have attracted considerable attention in academia and industry as a substitute for silicon-based devices such as liquid crystal displays and inorganic LEDs [1,2]. One advantage of OLEDs, as well as other organic electronic devices such as organic photovoltaics and organic thin film transistors (OTFTs) versus traditional technologies, include their ability to be fabricated through solution processing techniques, including spin-coating [3], drop casting [4,5], and ink-jet printing [6]. OTFTs have been found to be an integral component of next generation applications such as pixel modulators in active matrix OLED displays [7], radiofrequency tags [8], and even biosensors [9].

The organic semiconducting (OSC) layer is the primary focus of research in OTFT technology, as it governs the operation of the device. A wide variety of materials have been proposed and investigated over the years [10]. Anthracene, being the first organic compound used to study organic semiconductor conductivity in the 1950s–1960s, is still a promising molecule [11,12,13]. Researchers developed OTFTs that employed anthracene-based molecules with mobilities as high as 0.02 cm^2^ V^−1^s^−1^ (p-type) by 2003 [14,15,16]. Since 2013, over 150 derivatives have been synthesized, providing the basis for developing various structure–property–mobility relationships [10,17]. 2,6-Diphenyl anthracene (2,6-DPA) has afforded the highest p-type mobilities yet, with 14.8 cm^2^ V^−1^s^−1^ in OTFTs and 34.0 cm^2^ V^−1^s^−1^ in organic single-crystal transistors [18,19]. According to the variety of anthracene-derivatives implemented in devices, charge mobility may be traced from mainly three key factors: (1) The relative energy levels—the alignment of the semiconductor highest occupied molecular orbital (HOMO) and/or lowest unoccupied molecular orbital (LUMO) energy levels with the Fermi level of the source or drain electrodes defining the injection barriers; (2) the supramolecular arrangement of molecules in the solid-state—packing in either the herringbone or lamellar fashion, along with intermolecular distances, plays a significant role in charge transport ability; and (3) the thin film morphology—ordered stacking and dense grains with few boundaries and traps can reduce the obstruction for charge transport [10,20,21]. By examining the crystalline packing of anthracene-based derivatives via X-ray diffraction (XRD), we can gain an understanding of the intermolecular distances between conjugated centers of molecules arranged in a single-crystal. In general, the shorter the distance between π-orbital centers of molecules, the greater the charge mobility of an OSC will be in a device. For instance, relatively short intermolecular distances of 2.84–2.86 Å indicate strong π–π interactions, which likely contribute to the high charge mobility observed in devices containing 2,6-DPA [18,19]. Therefore, such an analysis gives us some insight into how such anthracene-derivatives arrange themselves in thin film. This facilitates the prediction of charge mobilities in an OSC device. Modifying the anthracene structure through coupling reactions can extend the π-electron system and tune the molecular packing, while simultaneously modifying the frontier molecular orbital energy levels or the thermal stability of the derivative—factors that are all crucial to the proper function of materials in devices [22,23].

Over the years, considerable effort has been devoted to synthesizing 2,6− and 2,6,9,10−substituted anthracene derivatives as they have seemingly afforded the highest mobilities; however, relatively few 9,10-functionalized derivatives have been reported [10]. Anthracene derivatives substituted at the 9,10-position challenge the regular herringbone stacking observed in 2,6-functionalized derivatives and tend to form a more overlapped lamellar structure. This is an advantageous propensity for charge transport, and an even better performance should be expected if closer molecular distance can be realized by more abundant and intensive intermolecular effects. In this study, we report several novel 9,10-substituted anthracene-based molecules, whereby we characterize their optical, electrochemical, and thermal properties to build a structure–property–mobility relationship for anthracene-based semiconductors. We also analyzed the materials by single-crystal XRD and incorporated the materials in OTFTs, facilitating the comparison between solid-state arrangement and charge mobility.

## 2. Results and Discussion

### 2.1. Synthesis of 9,10-Disubstiuted Anthracenes

**Figure 1:**
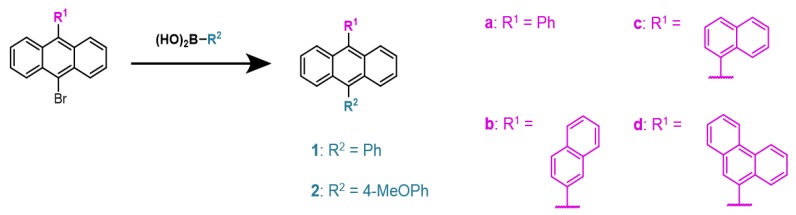
Synthesis of 9,10-disubstituted anthracenes (1a–c, 2a–d) via Suzuki-Miyura cross-coupling reactions.

A series of 9,10-disubstituted anthracenes (1a–c, 2a–d) were synthesized utilizing a palladium-catalyzed Suzuki-Miyura cross-coupling reaction starting from commercially available reagents, as shown in Figure 1. The general procedure for the aforementioned cross-coupling reactions employs tetrakis (triphenylphosphine) palladium (0) (Pd(PPh_3_)_4_) as the catalyst [24,25], coupling substituted bromoanthracenes with varying boronic acids in a degassed solvent mixture of toluene, ethanol, and water. The completion of coupling was determined through thin layer chromatography; conversion was achieved with heating of the mixtures overnight. The crude product was isolated by removal of the solvent and passing a dichloromethane (DCM) solution through a silica plug, separating the catalyst remnants. Sublimation of the crude materials provided a crystalline film of each desired product of high electronic purity. In some instances, recrystallization of the crude product in isopropanol produced a cleaner material for sublimation resulting in reduced yields. For analogue 2d, a N,N-dimethylformamide (DMF)/water solvent system was chosen to circumvent the solubility issues of the reagents.

### 2.2. Optical and Electrochemical Properties

Solutions of compounds 1a–c and 2a–d in DCM were characterized by UV-visible (UV-Vis) and photoluminescence (PL) spectroscopy; their corresponding maximum peak absorbance ($\lambda_{max}^{abs}$), energy gap ($E_{gap}$), and photoluminescence maximum peak emissions ($\lambda_{max}^{em}$) are all tabulated in Table 1 and their respective spectra can be found in the electronic supplementary information (ESI) (Figures S6–S12). The absorption profiles of each compound are similar to previously reported 9,10-disubstituted anthracenes, exhibiting four vibronic bands found between 325–425 nm corresponding to π–π* (S_0_ → S_1_) transitions of the anthracene core [26,27,28,29,30]. The minimal discrepancies between each of the $\lambda_{max}^{abs}$ suggests minimal orbital contribution from the exterior aryl groups, despite the alignment of the optical transition dipole moment along the short axis of these molecules. These observations can be rationalized through the twisted arrangement of the aryl groups in respect to the anthracene moiety [10,14,19,20,31,32,33,34,35]. With nearly identical absorption features, $E_{gap}$ were between 2.96–2.99 eV for each derivative.

Excitation of the compounds with the lowest energy $\lambda_{\max}^{\mathrm{abs}}$ produced emission spectra lacking mirror image quality with a blending of the fine structure. Stokes shifts varied between derivatives, where the largest shifts were 30 nm for both 2c and 2d, and the smallest shift was attributed to 2b of 7 nm. Solutions of each derivative were also excited with the next two higher energy $\lambda_{\max}^{\mathrm{abs}}$ and nearly identical emission profiles are observed, indicating similar relaxation pathways.

**Figure 2:**
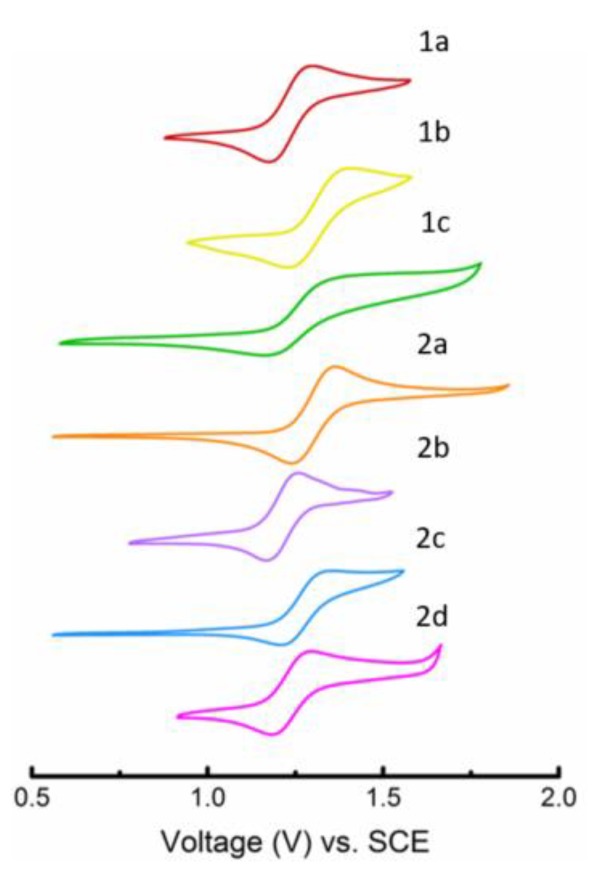
CV scans of 1a (red), 1b (yellow), 1c (green), 2a (orange), 2b (purple), 2c (blue), and 2d (magenta) in DCM.

In addition to studying the photophysical properties, cyclic voltammetry (CV) was performed on solutions of 1a–c and 2a–d in DCM (0.1 M *n*-Bu_4_NPF_6_ supporting electrolyte) to investigate their electrochemical behavior, as seen in Figure 2. A quasi-reversible oxidation process is observed for all derivatives with similar halfway oxidation potentials (*E*_1/2_). The HOMO energy levels (*E*_HOMO_) were estimated using the onset of the oxidation potentials [32,36,37,38,39,40]. The calculated *E_HOMO_* values were all around −5.59 eV and −5.73 eV, which is more negative than other derivatives reported [10,32,41].

The fact that all the CV data were similar indicates that the *E*_HOMO_ levels relative to the work function of the metal electrodes are similar as well, and therefore charge injection behavior should be similar throughout the materials. This *E*_HOMO_ level, in combination with the *E*_gap_, obtained from UV-Vis spectroscopy, would also suggest that all materials have similar stability to oxidation.

### 2.3. Thermogravimetric Analysis

**Figure 3:**
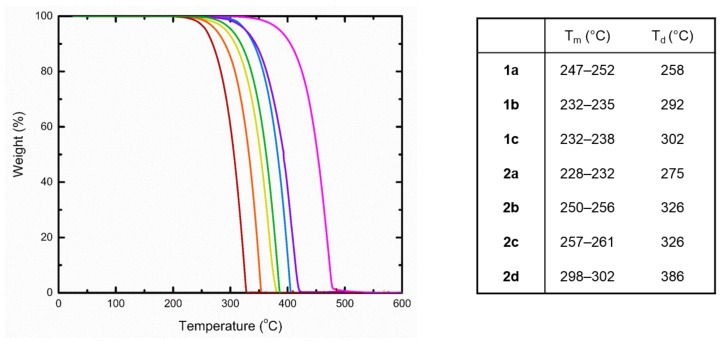
Thermogravimetric analysis curves from left to right: 1a (red), 1b (yellow), 1c (green), 2a (orange), 2b (purple), 2c (blue), and 2d (magenta) and their associated *T*_m_ and *T*_d_ (corresponding to 5% weight loss).

In addition to frontier molecular orbital energy levels, the thermal stability of a material is of considerable importance when attempting to develop a successful device. High temperatures associated with phase transitions (i.e., fusion) and decomposition pathways of these materials are ideal, so as to avoid structural changes and morphological arrangements of the thin films. Therefore, the melting points ™ and decomposition temperatures (*T*_d_) have been measured for compounds 1a–c and 2a–d and are tabulated in Figure 3. Unlike the optical properties, the choice in substituent played a significant role in the thermal properties. Thermogravimetric analysis (TGA) was performed on all compounds, where the decomposition temperature (*T*_d_) is determined at 5% weight loss. TGA was performed at a ramp heating rate of 5.0 °C min under a nitrogenous atmosphere. In general, molecules of 1 (258–302 °C) possessed lower *T*_d_ in comparison to their methoxy counterparts (275–386 °C). This trend is also recognized as the R^1^ group increases within each series, where 2d is superior with the highest *T*_d_ of 386°. In regard to *T*_m_, a similar pattern is apparent with 2, whereas the reverse is true for 1. These results may be a reflection of solid-state packing with stronger and/or more intermolecular interactions.

### 2.4. Single Crystal X-Ray Diffraction

**Figure 4:**
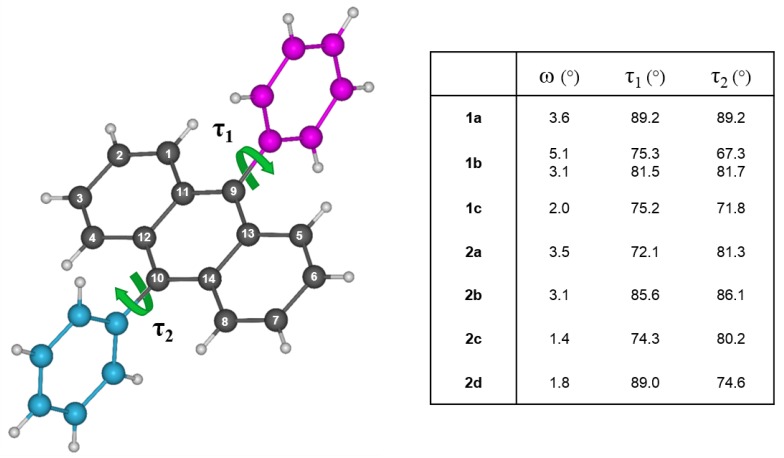
Molecular distortions depicted by the dihedral angle (ω) between intersecting planes from the outer blades of the anthracene backbone (C1–C4 and C5–C8) and the mean torsion angles (τ) between pendant aryl groups and anthracene unit. Compound 1b contains two unique molecules in the asymmetric unit.

To elucidate the structure–property relationship of the 9,10-disubstituted anthracenes, the molecular and solid-state structures were analyzed through X-ray crystallography. Single crystals were grown by train sublimation for each derivative and the crystallographic data are presented in Table S1. Between molecular entities, a number of commonalities are present with respect to their crystallographic features. For example, slight distortions are adopted along the backbone of the anthracene cores that disrupts its planarity. The degree of distortion is characterized by the dihedral angle (ω) created by intersecting planes bearing the terminal carbon atoms of the peripheral rings (Figure 4; i.e., C^1^–C^4^ and C^5^–C^8^), as well as based on the distance between the individual carbon atoms and the mean plane of the anthracene moiety (Table S2). The largest distortion is observed in 1b, which has two unique molecules in the asymmetric unit, with a dihedral angle of 5.1° and twelve out of the fourteen carbon atoms deviating from the mean plane by at least 0.03 Å for one of the molecules in the asymmetric unit. Interestingly, the other molecule experiences this distortion to a lesser extent with a dihedral angle of 3.1°, and half the number of carbon atoms deviating from the mean plane. Based on the family of compounds described here, it is apparent this distortion minimizes with the attachment of larger aryl groups and as such, may be resulting from a response to the packing arrangement of the molecules in the solid-state. Deviations in torsion angles (τ) between the pendent aryl substituents and the anthracene cores deviate from co-planarity with angles ranging from 67.3–89.43°, which can be attributed to steric interactions between the peri-hydrogen atoms of the anthracene core and the *ortho*-hydrogen atoms of the aryl substituents [32]. As a result, the twisting of the aryl substituents provides the foundation for self-assembly of the molecules in packing arrangements dominated by C–H···π interactions with anthracene units and aryl groups.

Understanding the molecular properties of these compounds can give us insights towards the supramolecular arrangements and the interactions between molecules. In regards to 1a, the near orthogonal phenyl substituents guide molecules in two-dimensional arrays parallel to the (010) plane, arranging in a lamellar-like structure (Figure S1). Where one phenyl moiety facilitates the C–H···π interactions (2.72–2.89 Å) with anthracene cores along the arrays, the other joins adjacent arrays through weaker C–H···π interactions (2.84 Å). A consequence resulting from the aforementioned interactions is the lack of superposition between anthracene units locked in a zig-zag pattern, significantly reducing π-orbital overlap between neighboring molecules. Consequently, the closest π-contacts are 3.79 and 3.92 Å within the arrays and 3.67 Å between arrays.

Replacement of the R^1^ phenyl substituent with a naphthalene moiety induces greater π-overlap amongst the anthracene cores, as shown in polycyclic hydrocarbons 1b and 1c. Compound 1b crystallizes with two unique molecules in the asymmetric unit, where spirals of alternating molecules run parallel to the *b*-axis connected by C–H···π interactions (2.80–2.84 Å), as seen in Figure S2. While the mean plane of the anthracene backbone belonging to one of the unique molecules is relatively planar to the (010) plane (i.e., perpendicular to the stacking axis; 88.6°), the other core deviates from the axis at an angle of 77.0°, rendering alternating π–π distances of 3.71 and 3.74 Å. This structural feature, in addition to the molecular distortions described earlier, could be a response to minimize the steric interactions arising from the protruding naphthyl substituents alongside of the spiral. More interesting is the interlocking of adjacent spirals through additional C–H···π interactions (2.89 Å) along the *c*-direction, providing shorter C–C contacts (3.46–3.60 Å) between neighboring anthracene units in two dimensions.

In contrast to 1b, molecules of 1c form slipped π-stacks parallel to the *b*-axis with the shortest π-contact of 3.72 Å between anthracene units (Figure S3). The attachment of the naphthyl group at the 2 position hinders superposition of the anthracene π-systems, similar to 1a, as C–H···π interactions (2.71–2.89 Å) lock anthracene domains in a criss-cross configuration. In addition, the naphthyl groups also facilitate C–H···π interactions (2.87 Å) that segregate π-stacks in staggered rows across the *b*-direction. Although interstack interactions are suppressed across this direction, adjacent stacks along the *a*-direction are closer together, providing additional but weaker π–π interactions (3.88 Å) between stacks.

**Figure 5:**
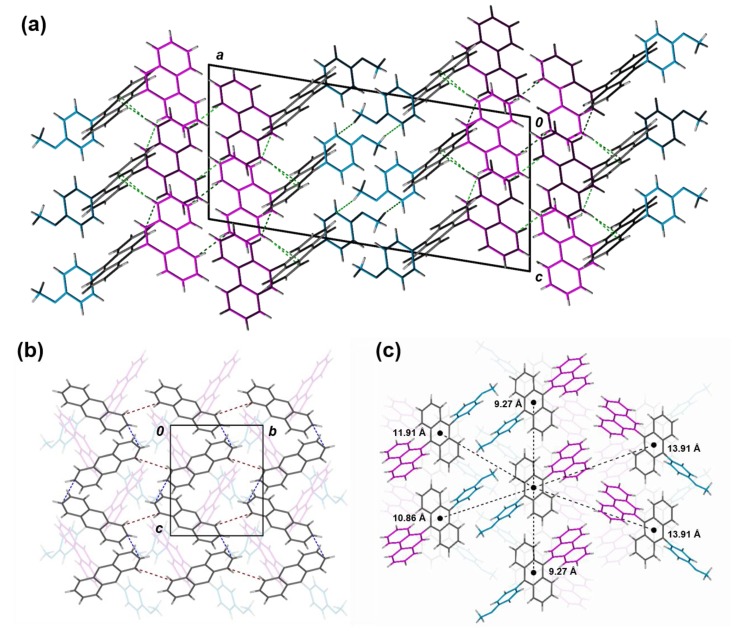
Slipped π-stacks of 2d viewed along the *a*-direction (a) and *b*-direction (b). C–H···π interactions between pendent substituents (R^1^ = magenta; R^2^ = blue) and anthracene cores are shown in green, while π–π contacts between anthracene units are shown in blue (intrastack) and red (interstack). (**c**) Distances between the centroids of neighboring molecules are illustrated between nearest π-stacks along the *c*-direction.

Inclusion of a methoxy group as the R^2^ substituent reinforces the π-stacking found in 2a, 2c, and 2d (Figures S4 and S5, Figure 4). For example, self-assembly of these derivatives is similar to 1c, such that staggered rows of stacked anthracene cores in a criss-cross arrangement, suggesting the size of the group influences this type of packing. As a result, the shortest π-contact within these stacks measures 3.63, 3.71, and 3.54 Å for 2a, 2c, and 2d, respectively (Figure 5). Interestingly, the addition of the various molecular substitutions did not have a large influence on the distance between the stacks of each row, thus retaining the weaker interstack π–π interactions of 3.90, 3.91, and 3.97 Å for 2a, 2c, and 2d, respectively. The same cannot be said for 2b, as hydrogen bonds in addition to C–H···π interactions lock in co-planarity between anthracene frameworks parallel to the (100) plane, ultimately disrupting the spiral motifs observed in 1b. The molecules are spaced out along their stacking axes in pairs to mitigate the steric repulsion from the protruding naphthalene groups, where the shortest C–C contact between anthracene moieties is 4.82 Å.

### 2.5. OTFT Performance

**Figure 6:**
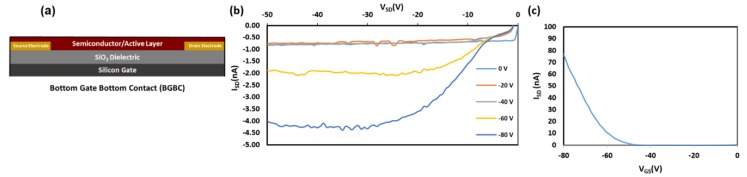
(**a**) Bottom gate bottom configuration used for organic thin film transistor (OTFT) integration of compounds 1b and 2a–d. (**b**) Characteristic output curve of fabricated OTFTs (1b). (**c**) Characteristic transfer curve of fabricated OTFTs (1b).

Compounds 1b and 2a–d were implemented into OTFTs by spin-coating the semiconductor onto Si/SiO_2_ substrates with prefabricated gold source-drain electrodes with channel widths, *W,* of 2000 μm and channel lengths, *L*, of 2.50 μm (Figure 6a). OTFT characteristics of compounds can be found in Table 2. All OTFTs were tested in air. Characteristic output and transfer curves of the integrated compounds are shown in Figure 6.

Compounds 1b and 2a,c–d exhibited field effect mobility, while compound 2b did not produce any field effect. On average, hole mobilities (*μ*_avg_) for compounds 1b and 2a,c–d were on the order of ≈3.2 × 10^−6^ cm^2^ V^−1^ s^−1^ with an average threshold voltage *(V*_T_) from −13 V to −43 V, and *I*_on/off_ ranging between 10^1^–10^2^. As expected, the OTFT performance is modest in comparison to previously reported examples, likely due to the relatively large π–π intermolecular distances as described above. All channel lengths were tested characterized (2.5, 5.0, 10.0, 20.0 μm). Only channel 2.5 μm length devices had observed field-effects. Annealing at 120 °C for 40 min was attempted to improve the performance, but resulted in reduced mobilities; agglomeration of the thin film was visible by microscope. Typical film thickness of these bottom gate bottom contact devices ranged from 157 to 1173 nm, significantly larger than the height of the Au electrodes (40 nm) relative to the SiO_2_ dielectric. These performance metrics are similar to other derivatives with comparable intermolecular packing distances, such as Silvestri et al.’s PA-P6d [41]. Wurthner et al.’s anthracene derivative also showed no field affect, whereby the π–π overlap of the face-to-face interactions of their molecule was approximately 50% and at a distance of 3.39 Å [42]. These results suggest that while 9,10-substitutions can act as handles to tune significantly tune the thermal stability, they also impart significant modifications on the solid-state arrangement, which can have detrimental effects on the OTFT performance [42]. Compounds 1a and 1c were not incorporated into devices based on the poor expected device performance, which would likely result from the large π–π distances obtained from single crystal diffraction (similar to device obtained using 2a and 2c).

## 3. Conclusions

Seven novel 9,10-functionalized anthracene-based molecules were synthesized, their optical, photoluminescent, electrochemical, and thermal characteristics were probed, and they were implemented as the active semiconducting layer in OTFTs. Based on this study, substitution of the 9,10-position proved to be an effective way to tune the thermal stability of the material, while having negligible effects on the frontier molecular orbital energy levels. Additionally, functionalization at the 9,10-position was found to strongly affect the solid-state arrangement, as determined through single crystal X-ray diffraction (XRD) analysis. These significant changes in solid-state structures resulted in OTFTs with modest performance, where the highest mobility obtained was on an order of 10^−6^ cm^2^ V^−1^ s^−1^ with a ***V_GS_*** of −80 V. These findings suggest that moiety functionalization strongly affects physicochemical properties such as melting point and decomposition temperature, yet has little effect on optical and electrochemical properties. These results further indicate that, towards improving anthracene-based OTFTs, it is wise to explore functionalization at various locations about the anthracene core rather than exclusively focusing on the 9,10-position. Future work will investigate the development of new molecules with such design strategies in mind to provide insight into their effect on solid-state engineering.

## 4. Materials and Methods

### 4.1. General Methods and Procedures

The reagents 9-bromo-10-phenylanthracene (Lumtec Corp., Taipei, Taiwan), 9-bromo-10-(naphthalene-1-yl)anthracene (Lumtec Corp., Taipei, Taiwan), 9-bromo-10-(phenanthrene-10-yl)anthracene (Lumtec Corp., Taipei, Taiwan), 9-bromo-10-(naphthalen-2-yl)anthracene (Lumtec Corp., Taipei, Taiwan), phenylboronic acid (Oakwood Products Inc., Estill, SC, USA), 4-methoxybenzeneboronic acid (Oakwood Products Inc., Estill, SC, USA), potassium carbonate (K_2_CO_3_) (Oakwood Products Inc., Estill, SC, USA), tetrakis(triphenylphosphine)palladium(0) (Pd(PPh_3_)_4_) (Strem Chemicals, Newburyport, MA, USA), toluene, N,N-dimethylformamide (DMF) (Caledon Laboratories Ltd., Georgetown, ON, Canada), and ethanol were commercially obtained and used as received. All solvents were ACS grade. All reactions were performed under an atmosphere of dry nitrogen. Melting points were taken using a Mel-Temp apparatus and are uncorrected. NMR spectra were run in CDCl_3_ solutions at room temperature on a Bruker 400 MHz spectrometer (Bruker, Billerica, MA, USA) and were referenced to the deuterated solvent peak. Film thickness measurements were performed with the Bruker DektakXT Profilometer (Bruker, Billerica, MA, USA). IR spectra were recorded on an Agilent Technologies Cary 630 FT-IR spectrometer. UV-Vis spectra were measured with a Varian Cary Series 6000 UV-Vis-NIR spectrophotometer (Agilent, Santa Clara, California, USA) and photoluminescence spectra were obtained using a Varian Cary Eclipse fluorescence spectrophotometer. UV-Vis and fluorescence spectra were measured in HQGC-grade DCM solutions with 1 cm precision quartz cuvettes. TGA analyses were performed in 70 µl alumina crucible using a TGA/DSC 1 Mettler Tolledo instrument (Mettler Tolledo, Columbus, Ohio, USA) under nitrogen gas with a heating rate of 5.0 °C min^−1^. Gas Chromatgraphy/Mass Sectrometry (GC/MS) was performed using Agilent 6890 GC (Agilent, Santa Clara, California, USA) coupled to Agilent 5975 MS. The inlet temperature was set 320 °C with a split ratio 50:1 at 1 µL injections. The initial oven temperature was 275 °C, held for 15 min, then ramped to 300 °C (40 °C /min) and held for 25 min. The column was HP-5MS (30 m × 250 µm × 0.25 µm), and its initial flow was 1.6 mL min^−1^. A toluene and 1,2-dichloroethane solvent mixture was used for all GC/MS experiments.

#### 4.1.1. Preparation of 9,10-diphenylanthracene (1a)

A bubbled-degassed solution of toluene, ethanol and water (1:0.25:0.15, 347 mL) was transferred to a mixture of 9-bromo-10-phenylanthracene (1.51 g, 4.53 mmol), phenylboronic acid (1.11 g, 9.10 mmol), K_2_CO_3_ (1.88 g, 13.60 mmol), and Pd(PPh_3_)_4_ (0.79 g, 0.68 mmol). The reaction was stirred for 16 h at 65 °C. After the reaction was cooled to room temperature, the solvent was removed in vacuo. The resulting solid was dissolved in a minimal amount of DCM, washed with a 1.0 M aqueous NaOH solution and subsequently with water and brine. The organic phase was dried with MgSO_4_, filtered through a silica plug and dried in vacuo, resulting in an off-white crude product. The crude product was recrystallized in isopropanol (Yield 0.42 g, 1.27 mmol, 28%) and further purified through sublimation at a temperature range of 205–215 °C under a pressure of 10^−3^ Torr with CO_2_ as a carrier gas, which afforded 1a as white crystals (Yield 0.26 g, 0.79 mmol, 17%). GC/MS reported an elution time of 6.760 min with abundance of 2.1 × 10^6^, and reported an M^+^ peak of 330.2 m/z compared to a prediction of 330.14 m/z. MP: 247–252 °C. ^1^H NMR (δ, 400 MHz, CDCl_3_): 7.69–7.73 (4H, m), 7.54–7.64 (6H, m), 7.48–7.52 (4H, m), 7.32–7.36 (4H, m). ^13^C NMR (δ, 100 MHz, CDCl_3_): 139.24 (2C), 137.26 (2C), 131.47 (4CH), 130.02 (4C), 128.55 (4CH), 127.61 (2CH), 127.11 (4CH), 125.13 (4CH). FT-IR (ν_max_): 3059 (w), 3032 (w), 1656 (w), 1598 (w), 1492 (w), 1439 (m), 1389 (m), 1369 (w), 1278 (w), 1193 (w), 1174 (w), 1158 (w), 1073 (m), 1025 (m), 999 (w), 942 (m), 923 (w), 856 (w), 845 (w), 767 (s), 748 (s), 732 (m), 700 (s), 661 (s) cm^−1^.

#### 4.1.2. Preparation of 9-phenyl-10-(1-naphthalenyl)-anthracene (1b)

Prepared analogously to 1a using 9-bromo-10-(naphthalene-1-yl)anthracene (1.50 g, 3.91 mmol), phenylboronic acid (0.95 g, 7.79 mmol), K_2_CO_3_ (1.62 g, 11.72 mmol), and Pd(PPh_3_)_4_ (0.68 g, 0.59 mmol) in 300 mL of the solvent mixture, yielding an off-white crude solid. Sublimation at a temperature range of 205–215 °C under a pressure of 10^−3^ Torr with CO_2_ as a carrier gas afforded 1b as white crystals (Yield 0.41 g, 1.08 mmol, 28%). GC/MS reported an elution time of 16.114 min with abundance of 2.1 × 10^7^, and reported an M^+^ peak of 380.2 m/z compared to a prediction of 380.16 m/z. MP: 232–235 °C. ^1^H NMR (δ, 400 MHz, CDCl_3_): 8.01–8.09 (2H, m), 7.70–7.76 (3H, m), 7.44–7.67 (9H, m), 7.30–7.35 (2H, m), 7.17–7.26 (5H, m). ^13^C NMR (δ, 100 MHz, CDCl_3_): 139.23 (1C), 137.63 (1C), 136.96 (1C), 135.15 (1C), 133.86 (1C), 133.76 (1C), 131.54 (2CH), 130.79 (2C), 130.08 (2C), 129.37 (CH), 128.58 (CH), 128.57 (CH), 128.37 (CH), 128.25 (CH), 127.66 (CH), 127.20 (2CH), 127.20 (2CH), 126.83 (CH), 126.42 (CH), 126.15 (CH), 125.75 (CH), 125.31 (2CH), 125.21 (2CH). FT-IR (ν_max_): 3047 (w), 2922 (w), 2853 (w), 1702 (w), 1655 (w), 1592 (w), 1561 (w), 1508 (w), 1438 (m), 1372 (m), 1255 (w), 1069 (w), 1028 (m), 1016 (w), 936 (m), 801 (m), 791 (w), 778 (s), 765 (s), 756 (s), 735 (m), 705 (s), 670 (m), 657 (s) cm^−1^.

#### 4.1.3. Preparation of 9-phenyl-10-(2-naphthalenyl)-anthracene (1c)

Prepared analogously to 1a using 9-bromo-10-(naphthalene-2-yl)anthracene (1.51 g, 3.94 mmol), phenylboronic acid (0.96 g, 7.88 mmol), K_2_CO_3_ (1.63 g, 11.80 mmol), and Pd(PPh_3_)_4_ (0.68 g, 0.59 mmol) in 287 mL of the solvent mixture, yielding an off-white crude solid. The crude product was recrystallized in isopropanol (Yield 0.39 g, 1.01 mmol, 26%) and further purified through sublimation at a temperature range of 155–185°C under a pressure of 10^−3^ Torr with CO_2_ as a carrier gas, which afforded 1c as white crystals (Yield 0.29 g, 0.76 mmol, 19%). GC/MS reported an elution time of 19.523 min with abundance of 5.1 x 10^6^, and reported an M^+^ peak of 380.2 m/z compared to a prediction of 380.16 m/z. MP: 232–238 °C. ^1^H NMR (δ, 400 MHz, CDCl_3_): 7.91–8.10 (4H, m), 7.71–7.76 (4H, m), 7.51–7.65 (8H, m), 7.29–7.35 (2H, m), 7.17–7.37 (4H, m). ^13^C NMR (δ, 100 MHz, CDCl_3_): 139.23 (1C), 137.42 (1C), 137.04 (1C), 136.76 (1C), 133.58 (1C), 132.92 (1C), 131.49 (2CH), 130.39 (1CH), 130.20 (2C), 130.06 (2C), 129.72 (1CH), 128.58 (2CH), 128.25 (1CH), 128.11 (1CH), 128.05 (1CH), 127.64 (1CH), 127.17 (2CH), 127.16 (2CH), 126.58 (1CH), 126.36 (1CH), 125.24 (2CH), 125.18 (2CH). FT-IR (ν_max_): 3051 (w), 1702 (w), 1655 (w), 1599 (w), 1561 (w), 1498 (w), 1438 (m), 1394 (m), 1370 (w), 1271 (w), 1244 (w), 1202 (w), 1176 (w), 1135 (w), 1070 (w), 1028 (m), 1017 (w), 1002 (w), 970 (w), 955 (w), 936 (m), 917 (w), 898 (w), 878 (w), 857 (w), 849 (w), 820 (m), 794 (w), 772 (m), 759 (s), 748 (s), 703 (s), 676 (m), 658 (s) cm^−1^.

#### 4.1.4. Preparation of 9-(4-methoxyphenyl)-10-phenylanthracene (2a)

Prepared analogously to 1a using 9-bromo-10-phenylanthracene (1.39 g, 4.17 mmol), 4-methoxybenzeneboronic acid (1.26 g, 8.29 mmol), K_2_CO_3_ (1.73 g, 12.52 mmol), and Pd(PPh_3_)_4_ (0.72 g, 0.62 mmol) in 318 mL of the solvent mixture, yielding an off-white crude solid. The crude product was recrystallized in isopropanol (Yield 0.76 g, 2.11 mmol, 51%) and further purified through sublimation at a temperature range of 150–180 °C under a pressure of 10^−3^ Torr with CO_2_ as a carrier gas, which afforded 2a as white crystals (Yield 0.45 g, 1.25 mmol, 30%). GC/MS reported an elution time of 11.715 min with abundance of 3.0 x 10^6^, and also reported an M^+^ peak of 360.2 m/z compared to a prediction of 360.15 m/z. MP: 228–232 °C. ^1^H NMR (δ, 400 MHz, CDCl_3_): 7.74–7.79 (2H, m), 7.68–7.73 (2H, m), 7.53–7.63 (3H, m), 7.47–7.50 (2H, m), 7.39–7.43 (2H, m), 7.31–7.37 (4H, m), 7.14–7.17 (2H, m), 3.97 (3H, s). ^13^C NMR (δ, 100 MHz, CDCl_3_): 159.18 (1C), 139.28 (1C), 137.08 (1C), 137.06 (1C), 132.53 (2CH), 131.48 (2CH), 131.25 (1C), 130.36 (2C), 130.06 (2C), 128.54 (2CH), 127.58 (CH), 127.19 (2CH), 127.10 (2CH), 125.10 (2CH), 125.05 (2CH), 114.02 (2CH), 55.54 (1CH_3_). FT-IR (ν_max_): 3067 (w), 3042 (w), 2999 (w), 2963 (w), 2936 (w), 2910 (w), 2841 (w), 1607 (m), 1575 (w), 1560 (w), 1513 (s), 1497 (m), 1463 (m), 1439 (m), 1409 (w), 1391 (m), 1370 (w), 1305 (w), 1284 (m), 1242 (s), 1190 (w), 1182 (m), 1176 (m), 1169 (m), 1158 (w), 1145 (w), 1136 (w), 1105 (m), 1071 (m), 1028 (s), 943 (m), 917 (w), 879 (w), 850 (m), 831 (s), 821 (m), 792 (m), 771 (s), 765 (s), 756 (s), 737 (m), 732 (m), 715 (m), 705 (s), 670 (s), 666 (s) cm^−1^.

#### 4.1.5. Preparation of 9-(4-(methoxyphenyl))-10-(1-naphthalenyl)anthracene (2b)

Prepared analogously to 1a using 9-bromo-10-(naphthalene-1-yl)anthracene (1.49 g, 3.89 mmol), 4-methoxybenzeneboronic acid (0.74 g, 4.86 mmol), K_2_CO_3_ (1.00 g, 7.29 mmol), and Pd(PPh_3_)_4_ (0.42 g, 0.36 mmol) in 191 mL of the solvent mixture, yielding an off-white crude solid. Sublimation at a temperature range of 155–185 °C under a pressure of 10^-3^ Torr with CO_2_ as a carrier gas afforded 2b as white crystals (Yield 0.76 g, 1.86 mmol, 48%). GC/MS reported an elution time of 51.288 min with abundance of 1.3 x 10^6^, and also reported a M^+^ peak of 410.2 m/z compared to a prediction of 410.1 m/z. MP: 250–256 °C.^−1^H NMR (δ, 400 MHz, CDCl_3_): 8.01–8.08 (2H, m), 7.79–7.81 (2H, m), 7.69–7.73 (1H, m), 7.57–7.59 (1H, m), 7.42–7.51 (5H, m), 7.31–7.35 (2H, m), 7.16–7.25 (6H, m), 3.99 (3H, s). ^13^C NMR (δ, 100 MHz, CDCl_3_): 159.23 (1C), 137.44 (1C), 137.01 (1C), 134.97 (1C), 133.86 (1C), 133.77 (1C), 132.60 (1CH), 132.59 (1CH), 131.24, 1 30.83 (2C), 130.43 (2C), 129.37 (1CH), 128.36 (1CH), 128.22 (1CH), 127.28 (2CH), 127.19 (2CH), 126.84 (1CH), 126.40 (1CH), 126.13 (1CH), 125.75 (1CH), 125.28 (2CH), 125.12 (2CH), 114.07 (1CH), 114.03 (1CH), 55.57 (1CH_3_). FT-IR (ν_max_): 3037 (w), 2958 (w), 2931 (w), 2837 (w), 1606 (m), 1561 (w), 1512 (m), 1509 (m), 1484 (w), 1459 (w), 1455 (m), 1444 (m), 1438 (m), 1405 (w), 1375 (m), 1302 (w), 1284 (m), 1244 (s), 1176 (m), 1148 (w), 1142 (w), 1107 (m), 1072 (w), 1026 (m), 1012 (m), 959 (w), 937 (m), 882 (w), 852 (w), 830 (m), 817 (w), 804 (s), 780 (s), 768 (s), 734 (m), 700 (m), 672 (m), 665 (m) cm^−1^.

#### 4.1.6. Preparation of 9-(4-methoxyphenyl)-10-(naphthalen-2-yl)anthracene (2c)

Prepared analogously to 1a using 9-bromo-10-(naphthalene-2-yl)anthracene (0.93 g, 2.44 mmol), 4-methoxybenzeneboronic acid (0.74 g, 4.86 mmol), K_2_CO_3_ (1.00 g, 7.24 mmol), and Pd(PPh_3_)_4_ (0.42 g, 0.36 mmol) in 318 mL of the solvent mixture, yielding an off-white crude solid. The crude product was recrystallized in isopropanol (Yield 0.39 g, 0.94 mmol, 39%) and further purified through sublimation at a temperature range of 155–180 °C under a pressure of 10^−3^ Torr with CO_2_ as a carrier gas, which afforded 2c as white crystals (Yield 0.33 g, 0.80 mmol, 33%). GC/MS reported an elution time of 26.614 min with abundance of 1.7 × 10^5^, and also reported an M^+^ peak of 410.2 m/z compared to a prediction of 410.17 m/z. MP: 257–261 °C. ^1^H NMR (δ, 400 MHz, CDCl_3_): 7.90–8.09 (4H, m), 7.71–7.80 (4H, m), 7.57–7.63 (3H, m), 7.41–7.45 (2H, m), 7.29–7.41 (4H, m), 7.15–7.19 (2H, m), 3.98 (3H, s). ^13^C NMR (δ, 100 MHz, CDCl_3_): 159.21 (1C), 137.23 (1C), 136.87 (1C), 136.81 (1C), 133.58 (1C), 132.91 (1C), 132.55 (2CH), 131.25 (1C), 130.40 (2C), 130.39 (1CH), 130.24 (2C), 129.74 (1CH), 128.24 (1CH), 128.10 (1CH), 128.05 (1CH), 127.26 (2CH), 127.15 (2CH), 126.56 (1CH), 126.34 (1CH), 125.21 (2CH), 125.09 (2CH), 114.05 (1CH), 114.04 (1CH), 55.55 (1CH_3_). FT-IR (ν_max_): 3035 (w), 2954 (w), 2932 (w), 2901 (w), 2835 (w), 1720 (w), 1702 (w), 1686 (w), 1655 (w), 1605 (w), 1560 (w), 1544 (w), 1509 (m), 1459 (w), 1438 (w), 1395 (w), 1296 (w), 1285 (w), 1271 (w), 1241 (s), 1202 (w), 1173 (m), 1136 (w), 1107 (w), 1033 (m), 1028 (m), 968 (w), 954 (w), 936 (m), 902 (w), 880 (w), 858 (w), 850 (w), 830 (w), 823 (m), 815 (m), 786 (w), 771 (m), 763 (s), 751 (s), 733 (m), 720 (w), 698 (w), 697 (w), 675 (m), 667 (m), 663 (m), 652 (w) cm^−1^.

#### 4.1.7. Preparation of 9-(4-methoxyphenyl)-10-(phenanthrene-10-yl)anthracene (2d)

A bubbled-degassed solution of DMF and water (9:1, 150 mL) was transferred to a mixture of 9-bromo-10-(phenanthrene-10-yl)anthracene (1.50 g, 3.46 mmol), 4-methoxybenzeneboronic (1.19 g, 7.83 mmol), K_2_CO_3_ (1.62 g, 11.74 mmol), and Pd(PPh_3_)_4_ (0.68 g, 0.59 mmol). The reaction was stirred for 16 h at 90 °C. After the reaction was cooled to room temperature, water (1.5 L) was added to the reaction. The resulting precipitate was filtered, washed with water, and dried. Sublimation at a temperature range of 220–245 °C under a pressure of 10^−3^ Torr with CO_2_ as a carrier gas, which afforded 2d as faint yellow crystals (Yield 1.26 g, 2.74 mmol, 79%). GC/MS reported an elution time of 51.288 min with abundance of 4.3 x 10^5^, and also reported an M^+^ peak of 460.3 m/z compared to a prediction of 460.3 m/z. MP: 298–302 °C. ^1^H NMR (δ, 400 MHz, CDCl_3_): 8.88–8.90 (2H, m), 7.92–7.95 (1H, m), 7.76–7.88 (4H, m), 7.65–7.71 (2H, m), 7.58–7.60 (2H, m), 7.46–7.54 (2H, m), 7.32–7.36 (3H, m), 7.18–7.28 (5H, m), 4.00 (3H, s). ^13^C NMR (δ, 100 MHz, CDCl_3_): 159.25 (1C), 137.56 (1C), 135.63 (1C), 134.80 (1C), 132.89 (1C), 132.61 (1CH), 132.59 (1CH), 131.92 (1C), 131.22 (1C), 130.89 (2C), 130.64 (1C), 130.57 (1C), 130.49 (2C), 130.15 (1CH), 128.93 (1CH), 127.71 (1CH), 127.34 (2CH), 127.21 (2CH), 127.10 (1CH), 127.04 (2CH), 126.84 (1CH), 125.39 (2CH), 125.19 (2CH), 123.00 (1CH), 122.88 (1CH), 114.09 (1CH), 114.04 (1CH), 55.56 (1CH_3_). FT-IR (ν_max_): 3060 (w), 3034 (w), 2953 (w), 2931 (w), 2899 (w), 2835 (w), 1606 (w), 1561 (w), 1510 (m), 1450 (w), 1438 (w), 1407 (w), 1390 (w), 1368 (w), 1310 (w), 1284 (m), 1281 (m), 1241 (m), 1172 (m), 1144 (w), 1108 (w), 1031 (m), 951 (w), 930 (w), 904 (w), 848 (w), 829 (m), 816 (w), 790 (w), 769 (s), 758 (m), 747 (s), 735 (s), 725 (s), 717 (m), 684 (m), 671 (m), 665 (m), 660 (m) cm^−1^.

### 4.2. Electrochemistry

Cyclic voltammetry was performed using a BASi Epsilon potentiostat employing a glass cell and platinum wires for working, counter, and pseudo-reference electrodes. The measurements were carried out on acetonitrile solutions (dried by J. C. Meyer solvent purification system and stored over 3 Å molecular sieves) containing 0.1 M tetrabutylammonium hexafluorophosphate (Oakwood) as supporting electrolyte with a scan rate of 100 mV/s. The experiments were referenced to the Fc/Fc+ redox couple of ferrocene at +0.475 V vs. saturated calomel electrode (SCE) [36,37,38]

### 4.3. Thermogravimetric Analysis

TGA analyses were performed in 70 ul alumina crucible using a TGA/DSC 1 Mettler Tolledo instrument under nitrogen gas with a heating rate of 5.0 °C min^−1^. All compounds, where the decomposition temperature (T_d_) is determined at 5% weight loss.

### 4.4. Crystallographic Characterization

Crystallographic data were collected from single crystals mounted on thin glass fibers using parabar oil and secured with clear nail polish. Data were collected on a Bruker Smart or Kappa APEX II single crystal diffractometer equipped with a graphite monochromator. Both instruments were equipped with a sealed tube Mo *K*α source (λ= 0.71073 Å), an APEX II CCD detector, and a dry compressed air-cooling system. All samples were cooled to 200 (2) K during data collection except for 2d, which remained at room temperature. Raw data collection and processing were performed with the APEX3 software package from Bruker [43]. Initial unit cell parameters were determined from 36 data frames from select ω scans. Semi-empirical absorption corrections based on equivalent reflections were applied [44]. Systematic absences in the diffraction data set and unit-cell parameters were consistent with the assigned space group. The initial structural solutions were determined using SHELXT direct methods [45] and refined with full-matrix least-squares procedures based on $F^{2}$ using SHELXL or ShelXle [46]. Hydrogen atoms were placed geometrically and refined using a riding model.

### 4.5. Electrical Characterization

Organic thin film transistors (OTFTs) were fabricated in a bottom gate bottom contact configuration by spin-coating the organic semiconductor onto Si/SiO_2_ substrates with prefabricated gold source-drain electrodes from Fraunhofer IPMS (*W* = 2000 μm, *L* = 2.5 μm). Prior to deposition, the substrates were washed by sequential sonication baths (5 min each) in soapy water, acetone, isopropanol, and dried with nitrogen followed by oxygen plasma for 15 min to clean and hydrolyze the surface. Substrates were then rinsed with water and isopropanol, then dried in nitrogen, before a 1.0 h surface treatment in 1% v/v octyltrichlorosilane (OTS) in toluene at 70 °C [47] Silane-treated substrates were washed with toluene and isopropanol and dried at 70 °C for 1h under vacuum. Then, 10 mg mL^−1^ solutions of compounds 1–5 were spin-coated by applying 1 mL drops of the respective solution onto the pre-patterned substrates and rotating at 2000 RPM. The substrates were then allowed to dry at 40 °C for 30 min under vacuum. Chloroform was used as the solvent to spin-coat material onto the substrate surface at 2000 RPM. All channels lengths were tested (2.5, 5.0, 10.0, 20.0 μm). All values were taken as an average value from a minimum of four devices. Characterization was performed in air. Electrical measurements were performed using a custom electrical probe station with a chamber allowing for controlled atmosphere, oesProbe A10000-P290 (Element Instrumentation Inc. & Kreus Design Inc., Richmond, BC, Canada) with a Keithley 2614B to control source-drain voltage (*V*_DS_), gate voltage (*V_GS_*), and measure source-drain current (*I*_DS_). V_DS_ was maintained at a constant −50 V, while *V*_GS_ was varied from −40 to −80 V to obtain measurements of *I*_DS_. From these measurements, saturation-region field-effect mobility, on/off current ratio, and threshold voltage were determined.

The general expression relating current to field-effect mobility and gate voltage in the saturation mode is given in Equation (1):

(1) $$I_{DS}=\frac{\mu C_{i}W}{2L}{\left(V_{GS}-V_{T}\right)}^{2}$$

where I_DS_ is the source-drain voltage, µ is the field-effect mobility of the material (electron mobility in this study), *C*_i_ is the capacitance, *W* is the width of the channel, *L* is the length of the channel, *V*_GS_ is the gate-source voltage, and *V*_T_ is the threshold voltage. To obtain a linear relation, the square root of Equation (1) is taken, giving Equation (2), so that the mobility and threshold voltage can be calculated directly from the slope and x-intercept of an $\sqrt{I_{DS}}\;vs\;V_{GS}$ curve, respectively.

(2) $$\sqrt{I_{DS}}=\sqrt{\frac{\mu C_{i}W}{2L}}\left(V_{GS}-V_{T}\right)$$

Finally, the on/off ratio is determined by Equation (3):

(3) $$On/Off\;Ratio\;=\frac{I_{on}}{I_{off}}$$

where *I*_on_ and *I*_off_ are the highest and lowest currents, respectively, measured in the characterized gate voltage range.

## Figures and Tables

**Table 1:** Electrochemical and optical characterization of compounds 1a-c and 2a-d.

	$E_{1/2}\;(V){}^{a}$	$E_{HOMO}\;(\mathrm{eV}){}^{b}$	$\lambda_{max}^{abs}\;(\mathrm{nm})$	$E_{gap}\;(\mathrm{eV}){}^{c}$	$\lambda_{max}^{em}\;(\mathrm{nm})$	Stokes Shift (nm)
**1a**	1.241	−5.68	342, 358, 376, 397	2.98	420, 435	23
**1b**	1.323	−5.73	341, 357, 376, 396	2.98	414, 430	18
**1c**	1.377	−5.73	341, 357, 376, 396	2.99	420, 435	24
**2a**	1.303	−5.69	339, 358, 376, 396	2.96	421, 431	25
**2b**	1.215	−5.59	343, 359, 377, 398	2.96	405, 430	7
**2c**	1.279	−5.68	343, 358, 377, 398	2.97	428	30
**2d**	1.237	−5.61	342, 358, 377, 397	2.97	427	30

a. Volts versus Saturated calomel electrode (SCE); b. $E_{HOMO\;}=-4.80\;eV-\left(E_{onset}^{ox}\;vs.\frac{Fc}{Fc^{+}}\right)$; c. $E_{gap}$ was calculated from the onset of the lowest energy absorbance peak.

**Table 2:** P-type OTFT testing summary*^a^* of compounds 1a–c and 2a–d.

	π–π Distance (Å)*^b^*	*I*_on/off_	*μ*_avg_ (cm^2^ V^−1^ s^−1^)	*μ*_max_ (cm^2^ V^−1^ s^−1^)	*V*_T,Avg_ (V)	*V*_T,max_ (V)
**1a**	3.67, 3.79, 3.92					
**1b**	3.46, 3.58, 3.60, 3.71, 3.74	10^2^	5.31 × 10^−6^	7.26 × 10^−6^	−37	−21
**1c**	3.72, 3.88					
**2a**	3.63, 3.90	10^2^	8.08 × 10^−7^	4.44 × 10^−6^	−41	−14
**2b** ^c^	4.82					
**2c**	3.71, 3.91	10^1^	1.48 × 10^−7^	1.91 × 10^−6^	−29	−13
**2d**	3.54, 3.97	10^2^	6.68 × 10^−6^	7.07 × 10^−6^	−43	−34

a. Field-effect observed at gate voltages varied from −80 to −40V; Channel length of 2.5 μm and electrode width of 2000 μm, where I_on/off_ order of magnitude of on/off current ratios, μ_avg_ = average hole mobility, μ_max_ = max hole mobility, V_T,Avg_ = average threshold voltage, and V_T,max_ = average threshold voltage. b. Closest π–π contacts between neighboring molecules determined through single crystal X-ray diffraction. c. OTFTs based on compound 2b exhibited no significant field effect.

## Supplementary Materials

The following are available online at https://www.mdpi.com/1996-1944/12/17/2726/s1, Figures S1–S5: Single crystal Xray diffraction views and interactions Figures S6–S12: UV-Vis absorption spectrum and emission spectra normalized for comparison for all compounds. Table S1. Crystallographic parameters for 1a–c and 2a–d. Table S2. Distances (Å) between the individual carbon atoms and the mean plane of the anthracene moiety.

## References

[1] Chang Y.L., Song Y., Wang Z., Helander M.G., Qiu J., Chai L., Liu Z., Scholes G.D., Lu Z. (2013). Highly efficient warm white organic light-emitting diodes by triplet exciton conversion. Adv. Funct. Mater..

[2] Wang Z.B., Helander M.G., Qiu J., Puzzo D.P., Greiner M.T., Hudson Z.M., Wang S., Liu Z.W., Lu Z.H. (2011). Unlocking the full potential of organic light-emitting diodes on flexible plastic. Nat. Photonics.

[3] Piliego C., Jarzab D., Gigli G., Chen Z., Facchetti A., Loi M.A. (2009). High Electron mobility and ambient stability in solution-processed perylene-based organic field-effect transistors. Adv. Mater..

[4] Bettinger C.J., Becerril H.A., Kim D.H., Lee B.L., Lee S., Bao Z. (2011). Microfluidic Arrays for rapid characterization of organic thin-film transistor performance. Adv. Mater..

[5] Chen H.Z., Shi M.M., Aernouts T., Wang M., Borghs G., Heremans P. (2005). A Novel organic N-Type material: Fluorinated perylene diimide. Sol. Energy Mater. Sol. Cells.

[6] Tian H., Han Y., Bao C., Yan D., Geng Y., Wang F. (2012). An asymmetric oligomer based on thienoacene for solution processed crystal organic thin-film transistors. Chem. Commun..

[7] Ma R.-Q., Hewitt R., Rajan K., Silvernail J., Urbanik K., Hack M., Brown J.J. (2007). Flexible active-matrix OLED displays: Challenges and progress. J. Soc. Inf. Disp..

[8] Glawe A., Eggerath D., Schäfer F. (2015). Printing versus Coating - What Will Be the Future Production Technology for Printed Electronics?. AIP Conference Proceedings.

[9] Boileau N.T., Melville O.A., Mirka B., Cranston R., Lessard B.H. (2019). P and N type copper phthalocyanines as effective semiconductors in organic thin-film transistor based DNA biosensors at elevated temperatures. Rsc Adv..

[10] Chen M., Yan L., Zhao Y., Murtaza I., Meng H., Huang W. (2018). Anthracene-based semiconductors for organic field-effect transistors. J. Mater. Chem. C.

[11] Xie H., Cheng C.Y., Li L., Deng X.Y., Yang K.K., Wang Y.Z. (2018). Integrating shape-memory technology and photo-imaging on a polymer platform for a high-security information storage medium. J. Mater. Chem. C.

[12] Kommandeur J. (1961). Photoconductivity in organic single crystals. J. Phys. Chem. Solids.

[13] LeBlanc O.H. (1961). Band Structure and Transport of Holes and Electrons in Anthracene. J. Chem. Phys..

[14] Quinn J.T.E., Zhu J., Li X., Wang J., Li Y. (2017). Recent progress in the development of n-type organic semiconductors for organic field effect transistors. J. Mater. Chem. C.

[15] Dong H., Fu X., Liu J., Wang Z., Hu W. (2013). 25th anniversary article: Key points for high-mobility organic field-effect transistors. Adv. Mater..

[16] Zhang X., Zhao G., Zhen Y., Tu Z., He P., Yi Y., Dong H., Hu W. (2015). The position effect of an ethynyl spacer on the carrier mobility of anthracene derivatives. J. Mater. Chem. C.

[17] Chaari M., Kelemen Z., Planas J.G., Teixidor F., Choquesillo-Lazarte D., Ben Salah A., Viñas C., Núñez R. (2018). Photoluminescence in: M -Carborane-Anthracene Triads: A combined experimental and computational study. J. Mater. Chem. C.

[18] Liu J., Dong H., Wang Z., Ji D., Cheng C., Geng H., Zhang H., Zhen Y., Jiang L., Fu H. (2015). Thin film Field-Effect transistors of 2,6-Diphenyl Anthracene (DPA). Chem. Commun..

[19] Liu J., Zhang H., Dong H., Meng L., Jiang L., Jiang L., Wang Y., Yu J., Sun Y., Hu W. (2015). High mobility emissive organic semiconductor. Nat. Commun..

[20] Yan L., Zhao Y., Yu H., Hu Z., He Y., Li A., Goto O., Yan C., Chen T., Chen R. (2016). Influence of heteroatoms on the charge mobility of anthracene derivatives. J. Mater. Chem. C.

[21] Li A., Yan L., Liu M., Murtaza I., He C., Zhang D., He Y., Meng H. (2017). Highly responsive phototransistors based on 2,6-bis(4-methoxyphenyl)anthracene single crystal. J. Mater. Chem. C.

[22] Payne M.M., Parkin S.R., Anthony J.E., Kuo C.C., Jackson T.N. (2005). Organic field-effect transistors from solution-deposited functionalized acenes with mobilities as high as 1 Cm2/V·s. J. Am. Chem. Soc..

[23] Liu J., Liu J., Zhang Z., Xu C., Li Q., Zhou K., Dong H., Zhang X., Hu W. (2017). Enhancing field-effect mobility and maintaining solid-state emission by incorporating 2,6-diphenyl substitution to 9,10-bis(phenylethynyl)anthracene. J. Mater. Chem. C.

[24] Zafar M.N., Mohsin M.A., Danish M., Nazar M.F., Murtaza S. (2014). Palladium catalyzed heck − mizoroki and suzuki − miyaura coupling reactions (Review). Russ. J. Coord. Chem..

[25] Smith G.B., Dezeny G.C., Hughes D.L., King A.O., Verhoeven T.R. (1994). Mechanistic studies of the suzuki cross-coupling reaction. J. Org. Chem..

[26] Wan Z., Qi W., Xing F., Li Y. (2017). Anthracene-Based Derivatives: Synthesis, Photophysical Properties and Electrochemical Properties. Chem. Res. Chin. Univ..

[27] Kim R., Lee S., Kim K.H., Lee Y.J., Kwon S.K., Kim J.J., Kim Y.H. (2013). Extremely deep blue and highly efficient non-doped organic light emitting diodes using an asymmetric anthracene derivative with a xylene unit. Chem. Commun..

[28] Wang Z.Q., Xu C., Wang W.Z., Duan L.M., Li Z., Zhao B.T., Ji B.M. (2012). High-color-purity and high-efficiency non-doped deep-blue electroluminescent devices based on novel anthracene derivatives. New J. Chem..

[29] Serevičius T., Komskis R., Adomėnas P., Adomėnienė O., Jankauskas V., Gruodis A., Juršėnas S. (2014). Non-symmetric 9, 10-diphenylanthracene-based deep-blue emitters with enhanced charge transport properties. Phys. Chem. Chem. Phys..

[30] Roberto P., Pietro T., Carlo S., Paolo P., Roberta D.A. (2015). Synthesis and Photophysical Properties of 9,10-Disubstituted Anthracenes. Sci. Res..

[31] Gidron O., Dadvand A., Wei-Hsin Sun E., Chung I., Shimon L.J.W., Bendikov M., Perepichka D.F. (2013). Oligofuran-containing molecules for organic electronics. J. Mater. Chem. C.

[32] Dadvand A., Sun W.H., Moiseev A.G., Bélanger-Gariépy F., Rosei F., Meng H., Perepichka D.F. (2013). 1,5-, 2,6- and 9,10-distyrylanthracenes as luminescent organic semiconductors. J. Mater. Chem. C.

[33] Usta H., Kim C., Wang Z., Lu S., Huang H., Facchetti A., Marks T.J. (2012). Anthracenedicarboximide-based semiconductors for air-stable, n-channel organic thin-film transistors: Materials design, synthesis, and structural characterization. J. Mater. Chem..

[34] Chen M., Zhao Y., Yan L., Yang S., Zhu Y., Murtaza I., He G., Meng H., Huang W. (2017). A unique blend of 2-fluorenyl-2-anthracene and 2-anthryl-2-anthracence showing white emission and high charge mobility. Angew. Chem. - Int. Ed..

[35] Dadvand A., Moiseev A.G., Sawabe K., Sun W.H., Djukic B., Chung I., Takenobu T., Rosei F., Perepichka D.F. (2012). Maximizing field-effect mobility and solid-state luminescence in organic semiconductors. Angew. Chem. - Int. Ed..

[36] Ding S.Y., Gao J., Wang Q., Zhang Y., Song W.G., Su C.Y., Wang W. (2011). Construction of covalent organic framework for catalysis: Pd/cof-lzu1 in suzuki-miyaura coupling reaction. J. Am. Chem. Soc..

[37] Aranzaes J.R., Daniel M.-C., Astruc D. (2006). Metallocenes as references for the determination of redox potentials by cyclic voltammetry—permethylated iron and cobalt sandwich complexes, inhibition by polyamine dendrimers, and the role of hydroxy-containing ferrocenes. Can. J. Chem..

[38] Li Y., Cao Y., Gao J., Wang D., Yu G., Heeger A.J. (2002). Electrochemical properties of luminescent polymers and polymer light-emitting electrochemical cells. Synth. Met..

[39] Dang M.T., Grant T.M., Yan H., Seferos D.S., Lessard B.H., Bender T.P. (2017). Bis(Tri-n-Alkylsilyl Oxide) silicon phthalocyanines: A start to establishing a structure property relationship as both ternary additives and non-fullerene electron acceptors in bulk heterojunction organic photovoltaic devices. J. Mater. Chem. A.

[40] Lohrman J., Zhang C., Zhang W., Ren S. (2012). Semiconducting carbon nanotube and covalent organic polyhedron-c60nanohybrids for light harvesting. Chem. Commun..

[41] Silvestri F., Marrocchi A., Seri M., Kim C., Marks T.J., Facchetti A., Taticchi A. (2010). Solution-processable low-molecular weight extended arylacetylenes: Versatile p-type semiconductors for field-effect transistors and bulk heterojunction solar cells. J. Am. Chem. Soc..

[42] Schmidt R., Göttling S., Leusser D., Stalke D., Krause A.M., Würthner F. (2006). Highly Soluble Acenes as semiconductors for thin film transistors. J. Mater. Chem..

[43] (2010). APEX Softward Suite v 2010.

[44] Blessing R.H. (1995). An empirical correction for absorption anisotropy. Acta Crystallogr. Sect. A: Found. Crystallogr..

[45] Hübschle C.B., Sheldrick G.M., Dittrich B. (2011). ShelXle: A Qt graphical user interface for SHELXL. J. Appl. Crystallogr..

[46] Sheldrick G.M. (2008). A short history of SHELX. Acta Crystallogr. Acta Crystallogr. Sect. A: Found. Crystallogr..

[47] Brixi S., Melville O.A., Boileau N.T., Lessard B.H. (2018). The influence of air and temperature on the performance of pbdb-t and p3ht in organic thin film transistors. J. Mater. Chem. C.

