# Supplementary material for: Developing 9,10-anthracene Derivatives: Optical, Electrochemical, Thermal, and Electrical Characterization
Source: Materials (Basel). 2019 Aug 26;12(17):2726. doi: 10.3390/ma12172726 (PMC6747803; doi:10.3390/ma12172726)
Supplement: Supplementary file 1 [file materials-12-02726-s001.pdf]

materials

Article

Developing 9,10-anthracene Derivatives: Optical, Electrochemical, Thermal, and Electrical Characterization

Mikhail Y. Vorona [1], Nathan J. Yutronkie [2], Owen A. Melville[1], Andrew J. Daszczynski [1,2], Kwame T. Agyei [1,2], Jeffrey S. Ovens [3], Jaclyn L. Brusso [2,*] and Benoît H. Lessard [1,*]

[1] Department of Chemical and Biological Engineering, University of Ottawa, 161 Louis Pasteur, Ottawa K1N 6N5, ON, Canada
[2] Department of Chemistry and Biomolecular Sciences, University of Ottawa, 150 Louis Pasteur, Ottawa K1N 6N5, ON, Canada
[3] X-Ray Core Facility, University of Ottawa, 150 Louis Pasteur, Ottawa K1N 6N5, ON, Canada
* Correspondence: benoit.lessard@uottawa.ca (B.H.L); jbrusso@uottawa.ca (J.L.B)

Received: 10 July 2019; Accepted: 19 August 2019; Published: 24 August 2019

Electronic Supporting Information

Contents (12 pages total)	
Crystallography	Page S2
UV-Vis Spectroscopy	Page S8

Table 1. Crystallographic parameters for 1a–c and 2a–d.

	**1a**	**1b**	**1c**	**2a**	**2b**	**2c**	**2d**
Empirical Formula	$C_{26}H_{18}$	$C_{30}H_{20}$	$C_{30}H_{20}$	$C_{27}H_{20}O$	$C_{31}H_{22}O$	$C_{31}H_{22}O$	$C_{35}H_{24}O$
Formula Weight, g/mol	330.40	380.46	380.46	360.43	410.48	410.48	460.54
Crystal System	monoclinic	monoclinic	orthorhombic	orthorhombic	monoclinic	orthorhombic	monoclinic
Space Group	$P\ 2_1/n$	$P\ 2_1/c$	$P\ b\ c\ a$	$P\ n\ a\ 2_1$	$C\ 2/c$	$P\ b\ c\ a$	$P\ 2_1/c$
a, Å	9.1529(3)	14.0381(11)	9.255(3)	10.183(4)	25.3308(7)	9.2982(2)	23.621(4)
b, Å	21.0161(7)	17.7977(15)	10.583(3)	9.321(3)	9.3156(2)	10.6427(3)	9.2709(16)
c, Å	9.9591(3)	16.9329(14)	41.703(11)	20.096(7)	21.9276(9)	43.6240(11)	11.2030(17)
α, °	90	90	90	90	90	90	90
β, °	111.162(1)	106.84 (1)	90	90	122.983(1)	90	99.230(11)
γ, °	90	90	90	90	90	90	90
V, Å^3	1786.23(10)	4049.2(6)	4084.6(19)	1907.4(12)	4340.4(2)	4316.94(19)	2421.6(7)
Z	4	8	8	4	8	8	4
T, K	200(2)	200(2)	200(2)	200(2)	200(2)	200(2)	296(2)
ρ_{calc}, g/cm^3	1.228	1.248	1.237	1.255	1.256	1.263	1.263
μ, mm^{-1}	0.069	0.071	0.070	0.075	0.074	0.075	0.074
$2\theta_{max}$, °	56.752	59.166	50.054	53.49	59.83	73.24	53.012
Total/Unique Reflections	29388/4453	60826/10672	23008/3617	41476/4037	47938/6027	52220/6656	18018/4968
Reflections $[I_o \geq 2\sigma(I_o)]$	3390	6990	2160	2817	5126	3620	2804
Parameters/Restraints	235/0	541/0	354/177	254/1	290/0	375/193	326/0
$R_1,\ wR_2\ [I_o \geq 2\sigma(I_o)]^a$	0.0426, 0.1100	0.0659, 0.1809	0.0766, 0.1420	0.0446, 0.0966	0.0481, 0.1311	0.0698, 0.1636	0.0551, 0.1277
Goodness of Fit	1.058	1.043	1.093	1.143	1.059	1.030	1.010

[a]Function minimized: $\sum w(F_o^2 - F_c^2)^2$. $R_1 = \sum\left||F_o| - |F_c|\right|/\sum|F_o|$ and $wR_2 = [\sum(F_o^2 - F_c^2)^2/\sum F_o^4]^{\frac{1}{2}}$

Table 2. Distances (Å) between the individual carbon atoms and the mean plane of the anthracene moiety.

	C1	C2	C3	C4	C5	C6	C7	C8	C9	C10	C11	C12	C13	C14
**1a**	0.03	0.06	0.02	0.01	0.01	0.04	0.04	0.00	0.08	0.03	0.03	0.01	0.02	0.01
**1b**[1]	0.02<br>0.03	0.03<br>0.02	0.06<br>0.03	0.03<br>0.04	0.03<br>0.02	0.06<br>0.04	0.03<br>0.01	0.03<br>0.04	0.04<br>0.00	0.04<br>0.01	0.05<br>0.00	0.03<br>0.01	0.01<br>0.01	0.03<br>0.03
**1c**	0.02	0.01	0.03	0.02	0.03	0.02	0.01	0.02	0.00	0.01	0.04	0.01	0.00	0.01
**2a**	0.04	0.02	0.05	0.05	0.02	0.04	0.02	0.03	0.01	0.03	0.01	0.00	0.02	0.05
**2b**	0.03	0.01	0.02	0.03	0.04	0.04	0.02	0.04	0.03	0.00	0.01	0.02	0.00	0.02
**2c**	0.01	0.02	0.00	0.00	0.01	0.02	0.03	0.01	0.01	0.01	0.00	0.00	0.03	0.02
**2d**	0.02	0.01	0.02	0.02	0.01	0.02	0.01	0.01	0.02	0.03	0.01	0.01	0.00	0.02

[1]Compound **1b** contains two unique molecules in the asymmetric unit.

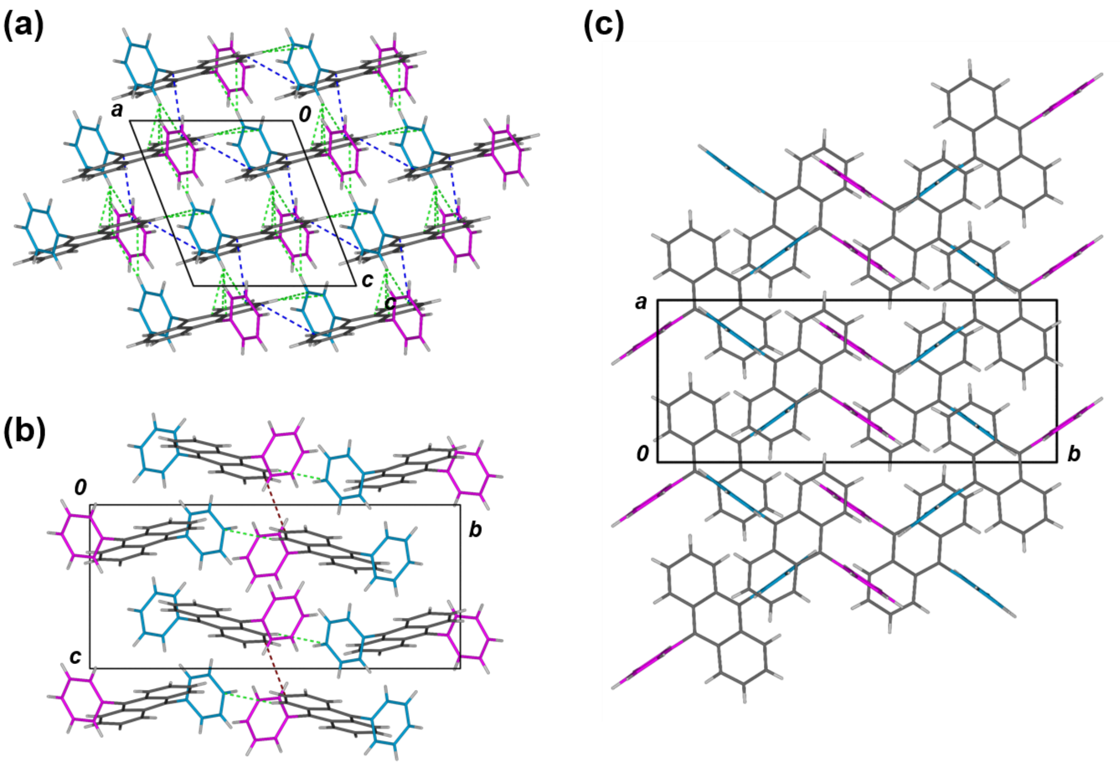

Figure S1. Two dimensional array of 1a parallel to the (010) plane (**a**) and its intercalating adjacent array viewed down *a*-direction (**b**) and *c*-direction (**c**). C–H···π interactions between pendent substituents (R^1 = magenta; R^2 = blue) and anthracene cores are shown in green, while π-π contacts between anthracene units are shown in blue (within array) and red (between arrays).

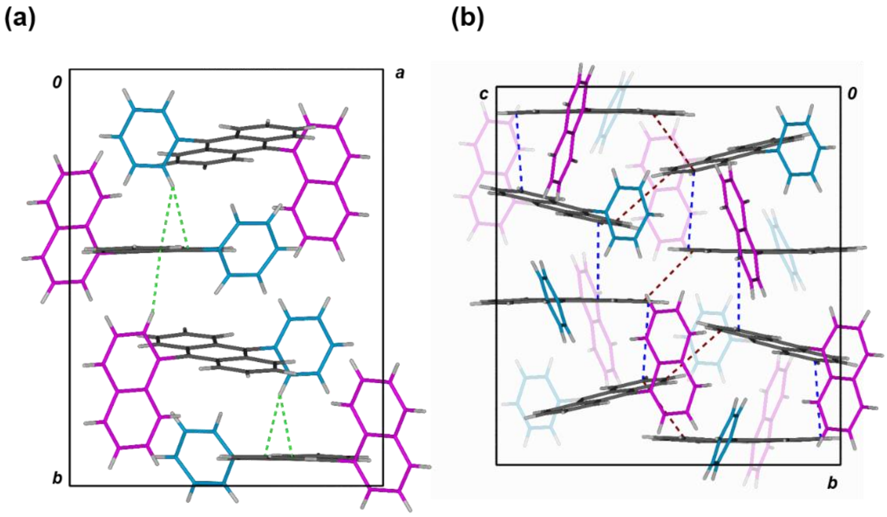

Figure S2. (**a**) Spiral of 1b along the *b*-direction. (**b**) Unit cell containing two interlocking spirals. C–H···π interactions between pendent substituents (R^1 = magenta; R^2 = blue) and anthracene cores are shown in green, while π-π contacts between anthracene units are shown in blue (within spiral) and red (between spirals).

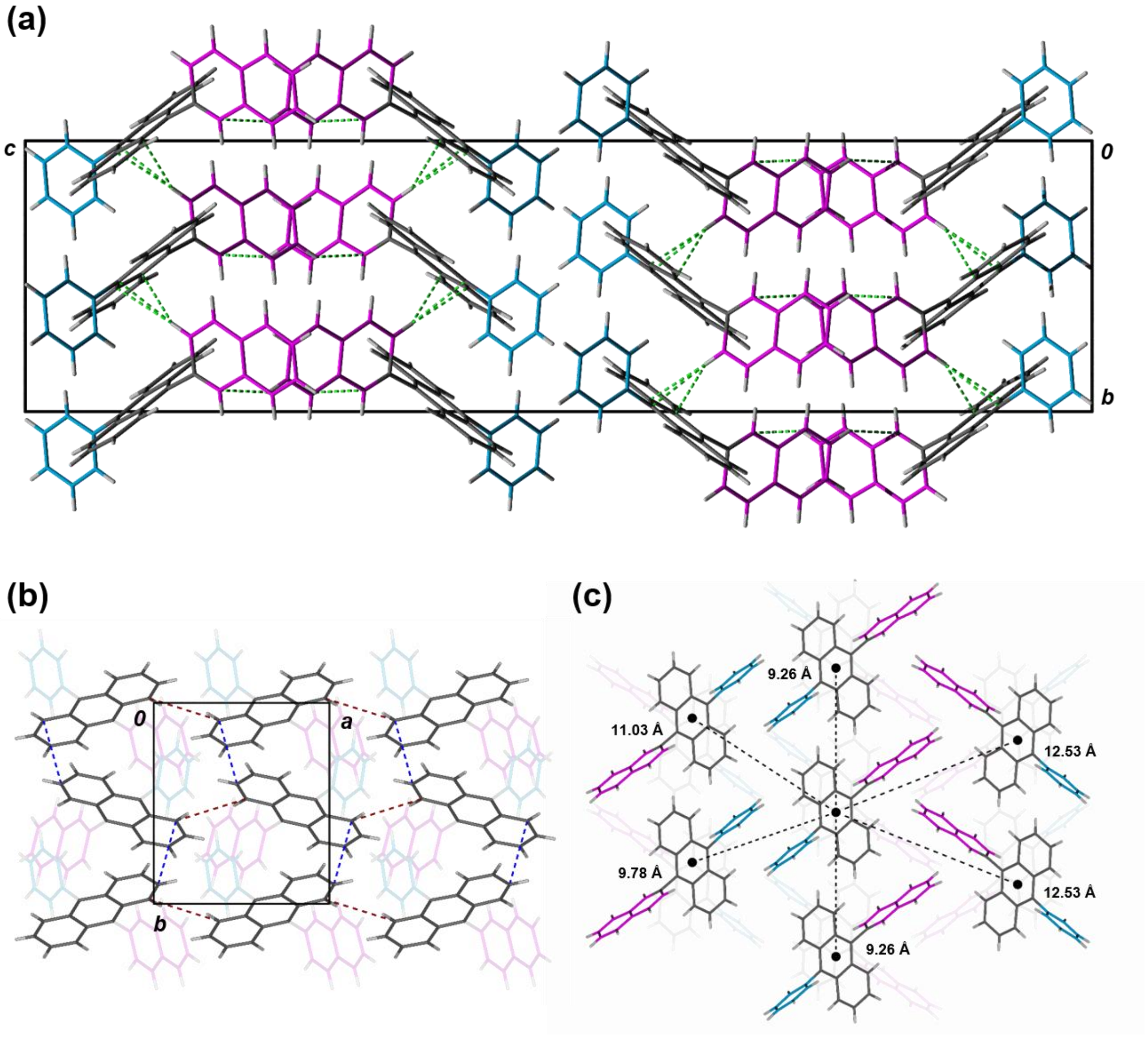

Figure S3. Slipped π-stacks of 1c viewed along the *a*-direction (**a**) and *c*-direction (**b**). C–H···π interactions between pendent substituents (R^1 = magenta; R^2 = blue) and anthracene cores are shown in green, while π-π contacts between anthracene units are shown in blue (intrastack) and red (interstack). (**c**) Distances between the centroids of neighbouring molecules are illustrated between adjacent π-stacks along the *c*-direction.

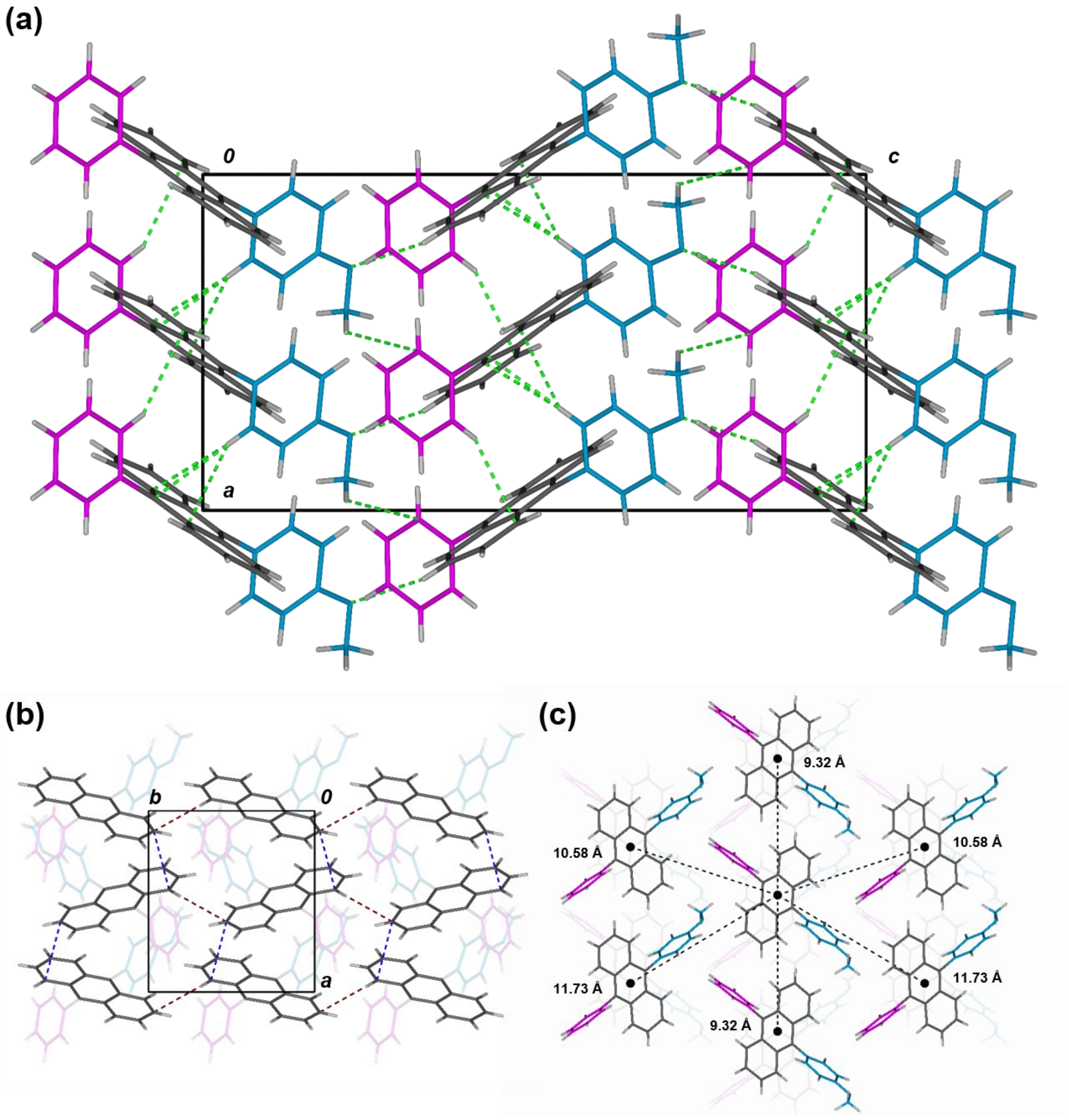

Figure 4. Slipped π-stacks of 2a viewed along the c-direction (**a**) and b-direction (**b**). C–H···π interactions between pendent substituents (R^1 = magenta; R^2 = blue) and anthracene cores are shown in green, while π-π contacts between anthracene units are shown in blue (intrastack) and red (interstack). (**c**) Distances between the centroids of neighbouring molecules are illustrated between adjacent π-stacks down the c-direction.

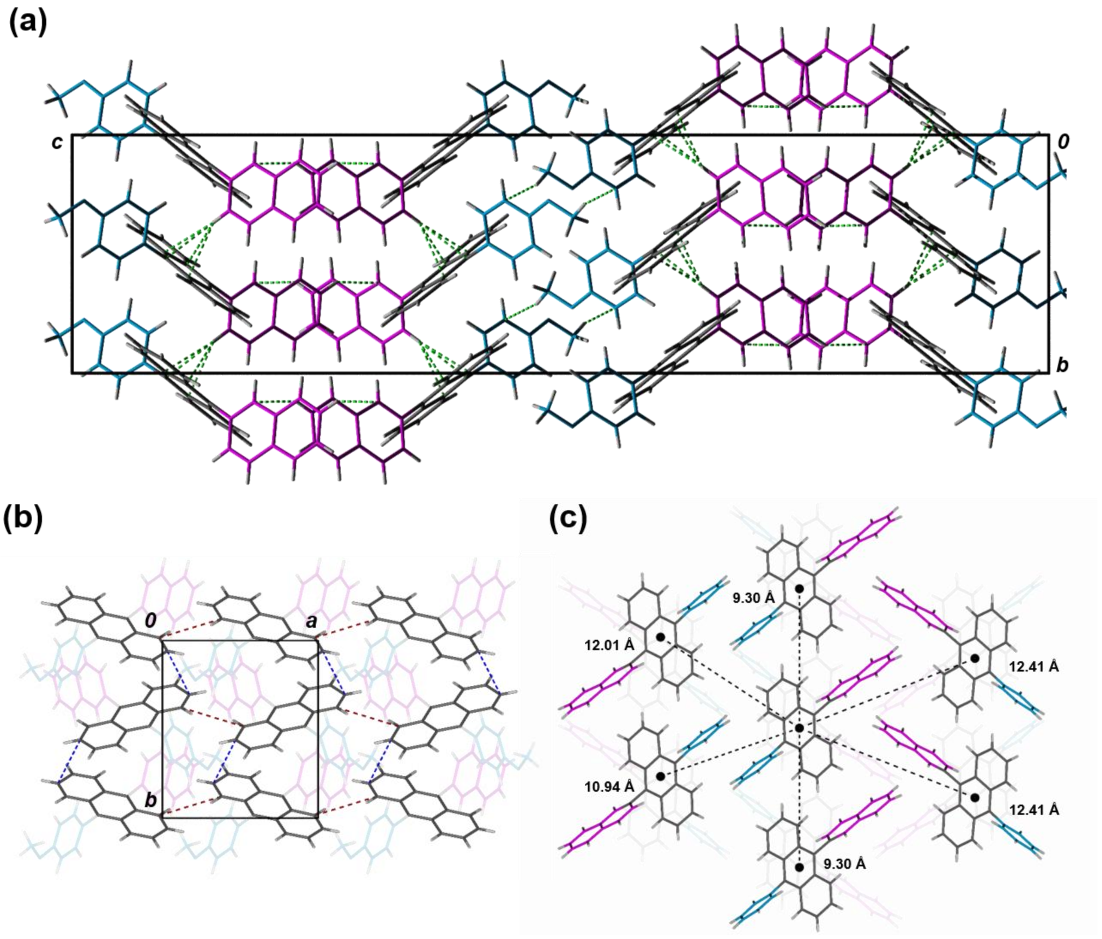

Figure S5. Slipped π-stacks of 2c viewed along the *c*-direction (**a**) and *a*-direction (**b**). C–H···π interactions between pendent substituents (R^1 = magenta; R^2 = blue) and anthracene cores are shown in green, while π-π contacts between anthracene units are shown in blue (intrastack) and red (interstack). (**c**) Distances between the centroids of neighbouring molecules are illustrated between adjacent π-stacks along the *b*-direction.

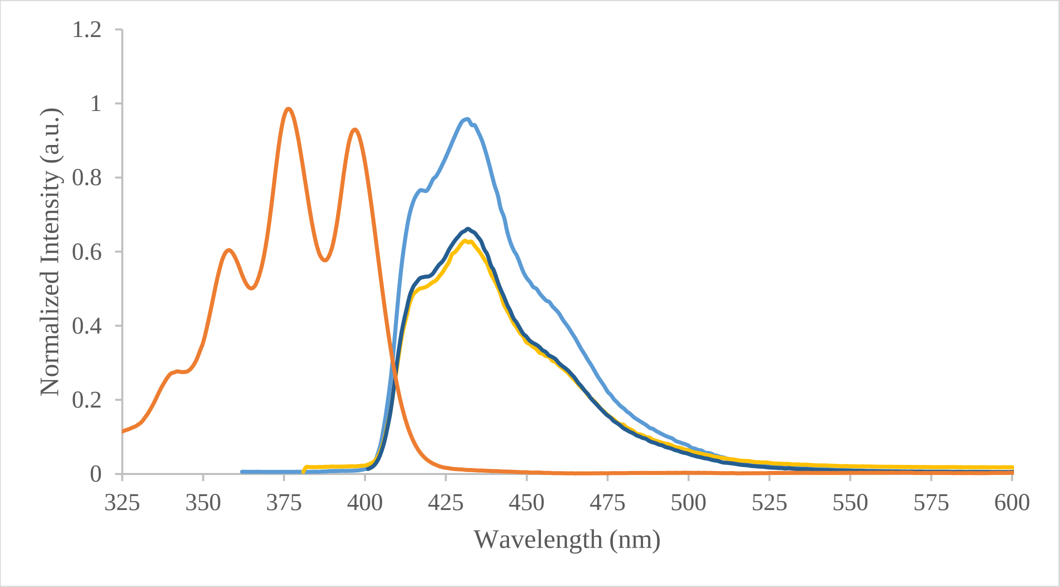

Figure S6. UV-Vis absorption (orange) spectrum and emission spectra with excitation at 357 nm (blue), 376 nm (yellow) and 396 nm (teal) of a DCM solution of **1a**. Absorption and emission spectra has been normalized for comparison.

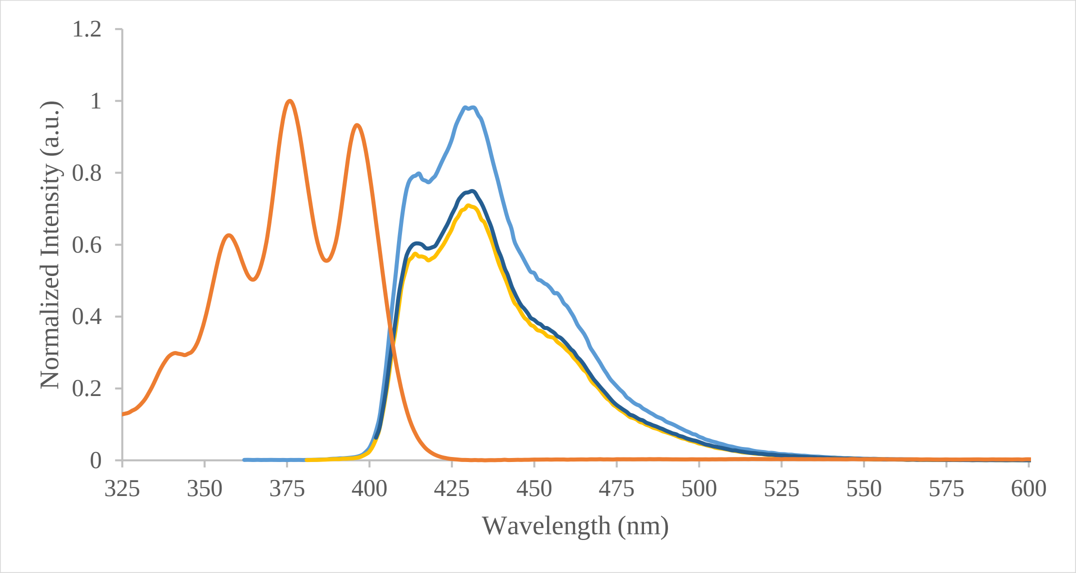

Figure 7. UV-Vis absorption (orange) spectrum and emission spectra with excitation at 357 nm (blue), 376 nm (yellow) and 397 nm (teal) of a DCM solution of 1b. Absorption and emission spectra has been normalized for comparison.

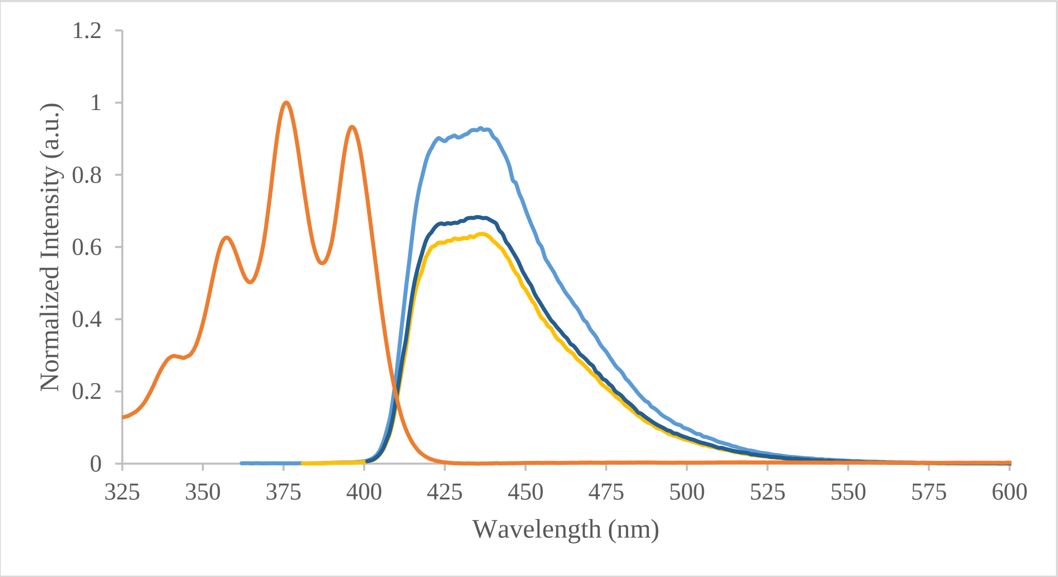

Figure 8. UV-Vis absorption (orange) spectrum and emission spectra with excitation at 357 nm (blue), 376 nm (yellow) and 396 nm (teal) of a DCM solution of **1c**. Absorption and emission spectra has been normalized for comparison.

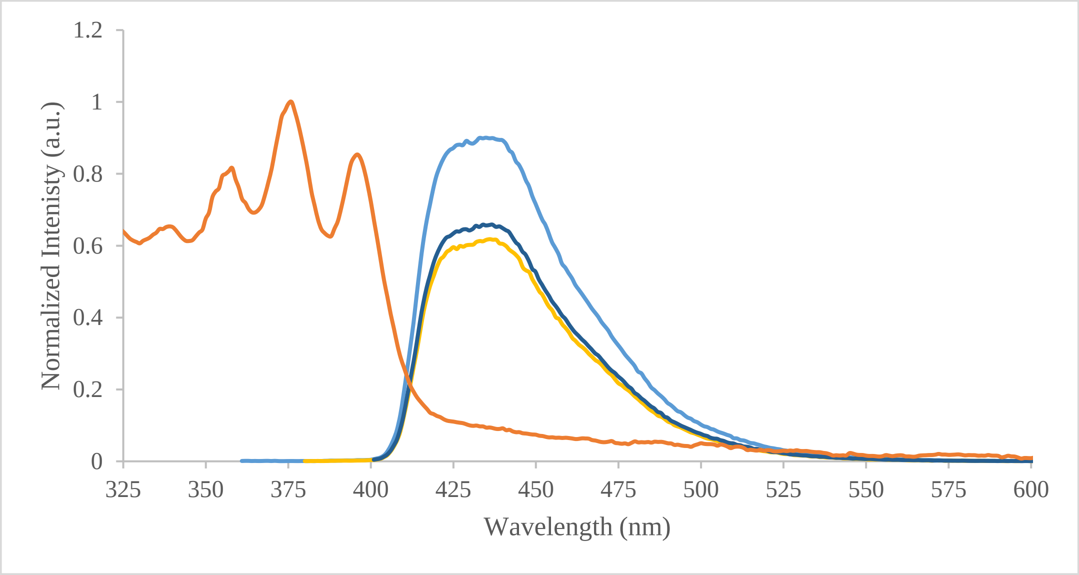

Figure S9. UV-Vis absorption (orange) spectrum and emission spectra with excitation at 356 nm (blue), 375 nm (yellow) and 396 nm (teal) of a DCM solution of 2a. Absorption and emission spectra has been normalized for comparison.

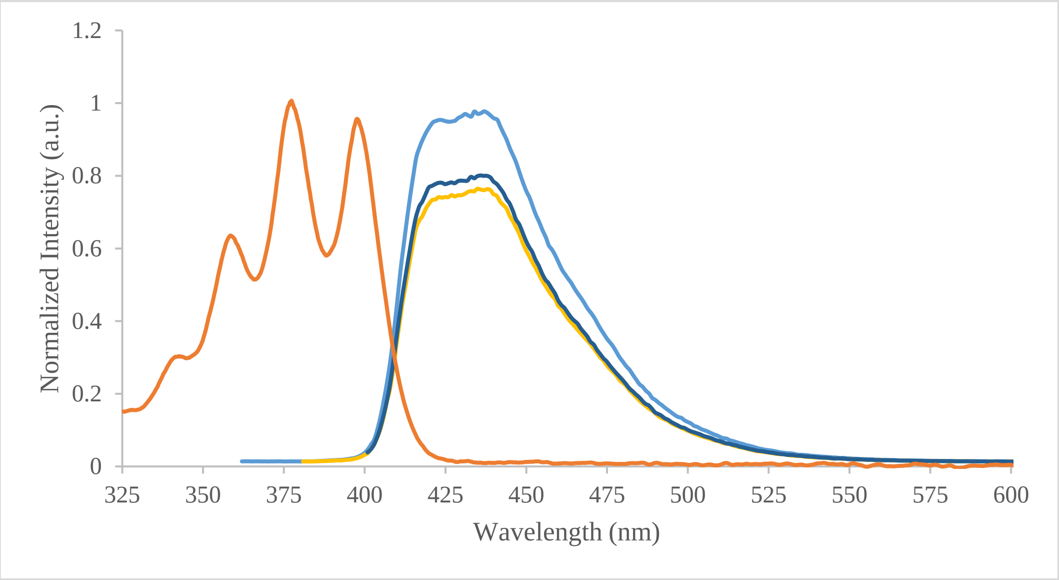

Figure 10. UV-Vis absorption (orange) spectrum and emission spectra with excitation at 357 nm (blue), 376 nm (yellow) and 396 nm (teal) of a DCM solution of **2b**. Absorption and emission spectra has been normalized for comparison.

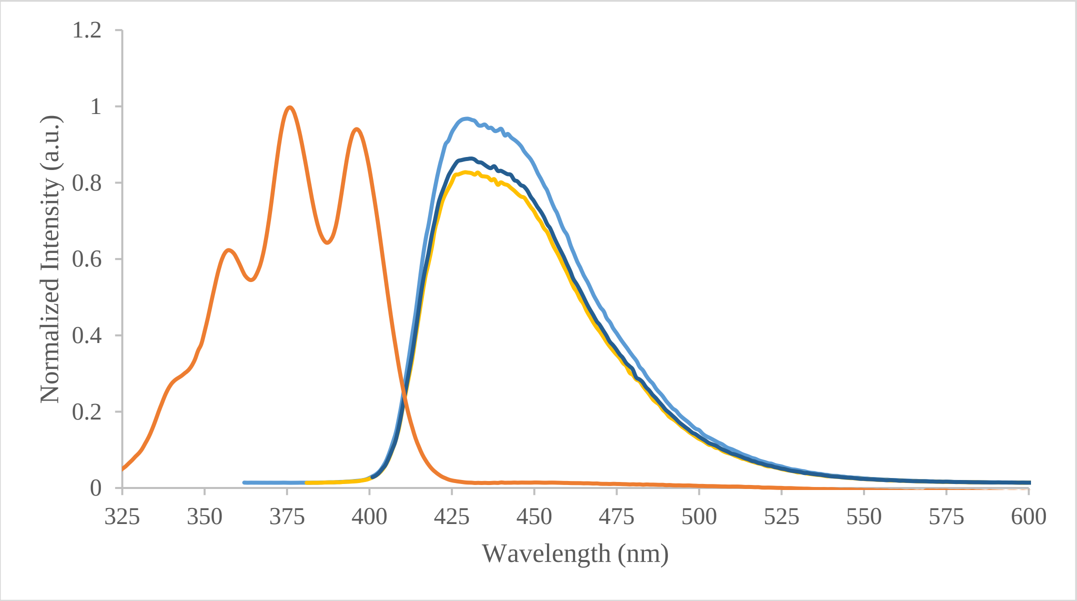

Figure 11. UV-Vis absorption (orange) spectrum and emission spectra with excitation at 357 nm (blue), 376 nm (yellow) and 396 nm (teal) of a DCM solution of **2c**. Absorption and emission spectra has been normalized for comparison.

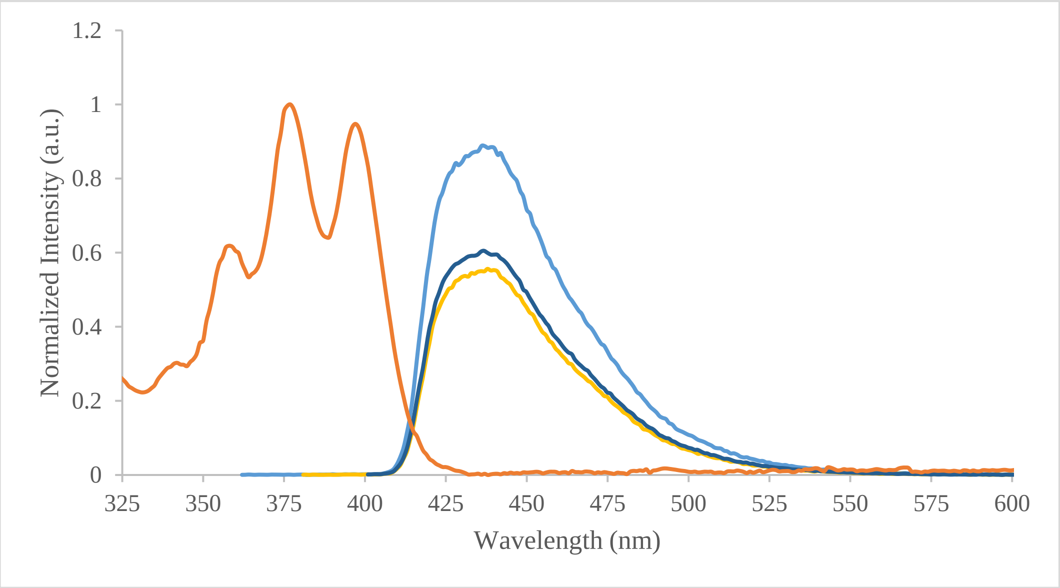

Figure 12. UV-Vis absorption (orange) spectrum and emission spectra with excitation at 357 nm (blue), 376 nm (yellow) and 396 nm (teal) of a DCM solution of 2d. Absorption and emission spectra has been normalized for comparison.
